# When endothelial cells go rogue

**DOI:** 10.15252/emmm.201505943

**Published:** 2015-11-27

**Authors:** Pei‐Yu Chen, Michael Simons

**Affiliations:** ^1^ Section of Cardiovascular MedicineDepartment of Internal MedicineYale Cardiovascular Research CenterNew HavenCTUSA; ^2^ Department of Cell BiologyYale University School of MedicineNew HavenCTUSA

**Keywords:** Cardiovascular System, Genetics, Gene Therapy & Genetic Disease, Vascular Biology & Angiogenesis

## Abstract

Endothelial‐to‐mesenchymal transition (EndMT) is a poorly understood phenomenon that leads to endothelial cells acquiring a variety of different mesenchymal fates. This results in a number of pathological consequences of considerable clinical significance in diseases ranging from cavernous malformations in the brain to tissue fibrosis, atherosclerosis, and cancer. Importantly, while there appears to be a number of different triggers activating EndMT, the final common pathway driving the transition appears to be the same.

A search for the Theory of Everything has been as much a feature of biological research as it has been a singular preoccupation of theoretical physicists. Lately, endothelial‐to‐mesenchymal (EndMT) transition has been making a concerted push to visibility, respectability, medical relevance, and, perhaps, a central place in a host of critical biological and pathophysiological processes.

EndMT was initially discovered as an essential step in heart development. Endothelial cells lining atrio‐ventricular canal and the outflow tract undergo EndMT and invade surrounding tissues to form the valves and septa of the heart (van Meeteren & ten Dijke, 2012). Further studies demonstrated EndMT occurrence in a broad spectrum of conditions including tissue fibrosis, cancer, and heterotopic ossification (van Meeteren & ten Dijke, 2012), as well as more recently in neointima formation (Chen *et al*, 2012; Cooley *et al*, 2014), cerebral cavernous malformations (CCM) (Maddaluno *et al*, 2013), and atherosclerosis (Chen *et al*, 2015) among others. The term “EndMT” encompasses a range of transformations—from the complete transdifferentiation of an endothelial cell into a mesenchymal cell to a number of intermediate states where cells retain some of their endothelial characteristics while acquiring distinctly mesenchymal features. This EndMT feature has a close parallel with the related and better understood process of epithelial‐to‐mesenchymal transition (EMT). Another parallel is that in both cases replacement of VE‐cadherin (EndMT) or E‐cadherin (EMT) with N‐cadherin is a key feature of mesenchymal transformation.

The direct pathological consequences of EndMT (whether partial or complete) include not only excessive production of mesenchymal cells (smooth muscle cells, fibroblasts, osteoblasts, adipocytes, etc.) but also abundant deposition of the extracellular matrix and increased thrombogenicity. Furthermore, evidence is now emerging that links EndMT directly to disease progression (Maddaluno *et al*, 2013; Chen *et al*, 2015). While considerable effort has gone into establishing that EndMT occurs and plays a role in a variety of pathologic conditions, the regulation of this process remains poorly understood.

One relatively well‐accepted concept is that the final common pathway leading to EndMT involves TGF‐β‐driven activation of transcription factors such as Snail, Slug, Twist, and zinc finger E‐box binding homeobox (ZEB) 1 and 2. These factors in turn upregulate mesenchymal gene expression. Yet molecular triggers of EndMT have not been well defined. Inflammation and mechanical stress are two conditions frequently associated with EndMT and a recent study demonstrated that both can activate TGF‐β signaling in the endothelium via downregulation of FGFR1 expression (Chen *et al*, 2015). The situation appears somewhat different in CCM, a common hemorrhagic vascular anomaly that can present both in sporadic and in familial autosomal dominant forms. Patients with CCM have loss‐of‐function mutations in one of the three CCM‐encoding genes (*CCM1*,* CCM2*, or *CCM3*) that result in formation of enlarged and irregular blood vessels with high propensity to bleeding leading to seizures and strokes (Fischer *et al*, 2013).

In an elegant series of studies in this issue of *EMBO Molecular Medicine*, Cuttano *et al* (2016) show that the loss of CCM1 activates the MEKK3‐MEK5‐ERK5 cascade that, in turn, upregulates KLF4. KLF4 then promotes TGF‐β/BMP signaling via production of BMP6. A deletion of KLF4 in the endothelial cell‐specific CCM1 knockout reduced the size and number of the lesions, prevented vascular leakage, and restored endothelial/astrocyte proximity in mice (Cuttano *et al*, 2016). Importantly, in a previous study, the same group demonstrated changes consistent with EndMT such as increased pSmad3 and N‐cadherin levels in human lesions due to mutations in CCM1 and CCM2 genes (Maddaluno *et al*, 2013).

The finding of the central role played by KLF4 in this disease setting is certainly intriguing. KLF4 is a zinc finger protein that functions as one of the reprogramming “Yamanaka factors” in pluripotent stem cell induction cocktails. It is also a shear stress‐inducible gene, a regulator of endothelial activation in response to pro‐inflammatory stimuli, a phenotypic modulator of smooth muscle cells and a protein intimately, if confusingly, involved with TGF‐β signaling (Yoshida & Hayashi, 2014). Similar to the strong induction of its expression seen in CCM lesions, KLF4 is also upregulated following the loss of endothelial FGF signaling input (Chen *et al*, 2014), another EndMT trigger, although its role in this form of EndMT has not been established. We thus have at least two different paradigms leading to EndMT activation: One is the loss of CCM gene‐mediated suppression of activation of the ERK5/KLF4 pathway, and the other is the inflammation‐ and shear stress‐driven loss of FGF signaling input that leads to increased expression of TGF‐β‐related genes. In both cases, the end result is Smad‐dependent activation of EndMT transcriptional program (Fig 1). It is very likely that other means of TGF‐β/BMP/Smad signaling activation also exist. Indeed Wnt/β‐catenin and Notch signaling pathways may well be involved as well.

**Figure 1:**
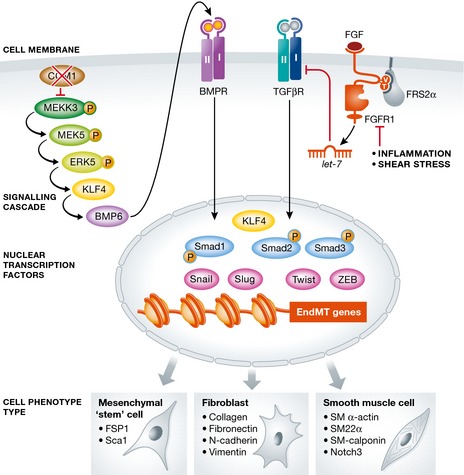
Two different signaling pathways leading to EndMT The loss of either CCM (left) or FGFR1 (right) leads to EndMT via two distinctly different pathways converging on activation of KLF4/Smad‐dependent transcription of the mesenchymal program.

This presents an interesting therapeutic dilemma. Presently, there are no treatments able to prevent development or reduce the size of existing CCMs nor are there effective approaches for prevention of EndMT in other disease settings, albeit TGF‐β/BMP inhibitors and drugs affecting signaling pathways potentiating TGF‐β/BMP such as Wnt/β‐catenin show promise in mice models (Maddaluno *et al*, 2013; Bravi *et al*, 2015; Chen *et al*, 2015). Yet, TGF‐β signaling has a number of beneficial effects such that systemic TGF‐β inhibition may not be desirable. Thus, the ability to selectively target various forms of EndMT, based on their specific signaling signatures and specific ways of activating the common TGF‐β/BMP/Smad pathway, may be of considerable interest.

EndMT is now emerging as a grand unifying concept bringing together a variety of initiating impulses that make endothelial cells “go rogue” and adapt mesenchymal features that play a central role in disease progression. The study from Cuttano *et al* (2016) is an important advance that further cements the central role of EndMT in a human disease entity and lays the groundwork for novel therapeutic developments.
